# Effects of Fatigue on Postural Sway and Electromyography Modulation in Young Expert Acrobatic Gymnasts and Healthy Non-trained Controls During Unipedal Stance

**DOI:** 10.3389/fphys.2022.782838

**Published:** 2022-02-09

**Authors:** Marcos Camargo da Silva, Cristiano Rocha da Silva, Felipe Fava de Lima, Jéssica Rodriguez Lara, Jackson Paiva Gustavson, Fernando Henrique Magalhães

**Affiliations:** ^1^School of Arts, Sciences and Humanities, Universidade de São Paulo, EACH-USP, São Paulo, Brazil; ^2^Biomedical Engineering Laboratory and Neuroscience Program, Universidade de São Paulo, EPUSP, PTC, São Paulo, Brazil

**Keywords:** postural control, balance, training, steadiness, sport

## Abstract

This study investigated whether expert acrobatic gymnasts respond differentially than their non-trained counterparts during a single-legged stance task performed before and after a protocol designed to induce fatigue in the ankle plantarflexor muscles in terms of (a) postural steadiness and (b) electromyography (EMG) activation. We hypothesized that neuromuscular adaptation due to training would lead to different behavior of center of pressure (COP) and EMG quantifiers after fatigue. Twenty eight female volunteers (aged 11 to 24 years) formed two groups: expert acrobatic gymnastics athletes (GYN, *n* = 14) and age-matched non-gymnasts [control (CTRL), *n* = 14]. Fatigue of the ankle plantarflexors (dominant leg) was induced by a sustained posture (standing on the toes) until exhaustion. Traditional COP parameters (area, RMS, mean velocity, and power spectrum at low and high frequency ranges) were obtained with a force plate, and time and frequency-domain EMG parameters were obtained by surface electrodes positioned on the *tibialis anterior*, *soleus*, *lateral gastrocnemius*, *medial gastrocnemius*, *vastus lateralis*, *biceps femoris*, *spinal erector* and *rectus abdominis* muscles. The main results showed that fatigue induced a significant increase in postural oscillations in the ML axis (including RMS, velocity and frequency components of the power spectrum), with no significant effects in the AP axis. In terms of postural sway parameters (i.e., COP quantifiers), no superior balance stability was found for the GYN group as compared to CTRL, irrespective of the fatigue condition. On the other hand, the modulation of EMG parameters (in both time and frequency domains) indicated that expert acrobatic gymnastics athletes (as compared to healthy untrained matched controls) used different neuromuscular control strategies to keep their postures on single-legged quiet standing after the fatiguing protocol. The present results improve our knowledge of the mechanisms behind the interplay between fatigue and postural performance associated with the neuromuscular adaptations induced by sport practice. The design of gymnastics training might consider strategies aimed at improving the performance of specific muscles (i.e., *tibialis anterior*, *soleus*, *biceps femoris*, *spinal erector*) for which particular activation patterns were used by the acrobatic gymnastics to control single-legged quiet standing.

## Introduction

The control of body balance (i.e., quiet stance) is regulated by the postural control system, which needs to integrate information from visual, vestibular and somatosensory sources to generate adequate motor responses that keep the body still (Winter, 1995; Peterka, 2002). Assessments of balance ability are routinely performed with the use of a force plate that tracks the location of the center of pressure (COP), especially when postural deviations are small so that the body might be simplified as an inverted pendulum model, thereby reflecting the so-called ankle strategy (Peterka, 2003).

Some studies have pointed out the practice of sports such as gymnastics promotes efficient stimuli in generating neuromuscular adaptations, which seems to be associated with intense and regular practice of complex motor skills, including high demand for production and torque control around the ankle joint. Specifically, ankle plantarflexors are traditionally considered the most important muscle group engaged in the maintenance and control of postural balance (Rothwell, 1994; Winter, 1995), and hence it is plausible that increased performance of these muscles (e.g., due to long-term practicing) is accompanied by improvements in postural steadiness. In this direction, some studies have shown that trained gymnasts exhibit superior balance ability as compared to untrained participants or athletes involved in sports activities with different characteristics (Debu and Woollacott, 1988; Carrick et al., 2007; Andreeva et al., 2021). However, this issue remains controversial, as other studies showed no significant differences in balance ability between gymnasts and controls under certain conditions. Indeed, the literature indicates that such a “superior balance ability” is influenced by a variety of factors such as task specificity (Vuillerme et al., 2001a; Asseman et al., 2004, 2008; Isableu et al., 2017), attentional demand (Vuillerme and Nougier, 2004), level of expertise (Opala-Berdzik et al., 2021), sensory condition (Vuillerme et al., 2001a; Asseman et al., 2005, 2008; Garcia et al., 2011), anthropometric characteristics (Opala-Berdzik et al., 2018, 2021), age (Garcia et al., 2011), sex (Milosis and Siatras, 2012) and the parameter of postural sway being analyzed (Isableu et al., 2017; Opala-Berdzik et al., 2018). To the best of our knowledge, the majority of these studies involved artistic gymnasts, although rhythmic and acrobatic gymnastics have also been explored, with somewhat similar results (Calavalle et al., 2008; Opala-Berdzik et al., 2018, 2021).

The performance of acrobatic gymnastics focuses on static and dynamic routines which demand high levels of strength, stability, flexibility and agility, and hence training programs are designed to enhance these features thereby improving overall performance. Additionally, practicing also focuses on the presentation of specific routines in which the athletes work together in an attempt to have their performance well ranked by referees. Consequently, the duration, frequency, and intensity of the training sessions are high, and often begin in early childhood. Therefore, one of the main issues coaches have to deal with concerns the deleterious effect of fatigue (that occurs during long and intense training sessions) on the athletes’ performance. In particular, the impact of fatigue on standing balance is of great importance as the overall stability during the static and dynamic routines are highly associated with the athletes’ performance (i.e., observed by referees during competitions). In this vein, fatigue has been considered of particular importance to sports-related injury risk and performance, and the negative effect of neuromuscular fatigue on postural control has been extensively reported in a wide variety of conditions and for different populations (Paillard, 2012). Traditionally, many studies have shown increased postural oscillations (and hence decreased steadiness) during quiet standing after the performance of a fatiguing exercise, especially involving the ankle musculature (Gribble and Hertel, 2004; Boyas et al., 2011, 2013; Bisson et al., 2012).

Surface electromyography (sEMG) has been widely used to evaluate neuromuscular adaptations as a function of fatigue (Rainoldi et al., 2008; Contessa et al., 2009; Soo et al., 2009; Dideriksen et al., 2010), and also to better understand the mechanisms associated with muscle activation during postural control (Magalhaes and Goroso, 2009; Mello et al., 2013). The fatigue-related increase in time domain parameters of the sEMG signals (i.e., amplitude, RMS) during sustained contractions has been attributed to an increase in neuromuscular activation, promoting greater recruitment of motor units to compensate for the saturation of fibers that are already fatigued, avoiding the immediate failure of the system (De Luca, 1997). On the other hand, fatigue-related changes on sEMG frequency-domain parameters [i.e., increase in low frequency components and decrease in high frequency components (Merletti et al., 1991; Lowery et al., 2002)] have been attributed to two main mechanisms: (1) peripheral changes, especially the decrease in the conduction velocity of muscle fiber action potentials and; (2) central changes including increased synchronism in the firing of motor units along with the recruitment of new motor units (Mathur et al., 2005).

However, despite the large number of studies mentioned above, to the best of our knowledge, no study has addressed the putative effects of neuromuscular adaptations resulting from acrobatic gymnastics training on the behavior of the postural control system in the presence of muscle fatigue. Additionally, presumed neuromuscular mechanisms reflected by changes in time and frequency-domain parameters of the EMG signal recorded during postural tasks (and its fatigue-related effects) remain unexplored. Such an investigation has the potential to provide insights that might be useful for the design of gymnastics training strategies, besides improving our knowledge on the mechanisms behind the interplay between fatigue and postural performance associated with the neuromuscular adaptations induced by sport practice. More specifically, a deeper understanding of the long-term effects of gymnastics training on postural control, together with information on how the neuromuscular system of experienced athletes adapt to overcome the deleterious effect of fatigue on postural stability might help coaches to focus on specific training strategies (such as strength/endurance/control training of specific muscle groups) that can potentially benefit their athletes in improving performance and/or reducing injury risk.

Therefore, the objective of this study was to investigate the effects of fatigue and acrobatic gymnastic expertise on the neuromuscular and behavioral performance of a unipedal quiet stance task. So, we assessed whether expert acrobatic gymnasts respond differentially than their non-trained counterparts during a single-legged stance task (performed before and after a protocol designed to induce fatigue in the ankle plantarflexors muscles) in terms of (a) postural steadiness and (b) EMG activation. The single-legged postural task was chosen because it is a posture commonly adopted in the practice of acrobatic gymnastics (present in the punctuation code considered by referees in competitions). The hypothesis addressed in the present study was that neuromuscular adaptation due to gymnastics training would lead to different behavior of postural sway and EMG quantifiers (acquired during unipedal quiet stance) in trained gymnasts submitted to neuromuscular fatigue of the plantarflexor muscles as compared to healthy untrained controls. More specifically, we expected that fatigue would reduce postural stability (as assessed by COP parameters) and change EMG activation patterns (increasing time-domain and decreasing frequency-domain parameters) in both expert acrobatic gymnasts and their non-trained counterparts; and that these changes would be more pronounced in the non-trained group, as the gymnasts would putatively have developed training-induced adaptation mechanisms that would help them in dealing with the deleterious effects of fatigue on postural control.

## Materials and Methods

### Participants

The experiments were carried out in 28 female volunteers, aged between 11 and 24 years. Two groups were formed: acrobatic gymnastics athletes (GYN, n = 14) and non-gymnasts [control (CTRL), *n* = 14]. Participants’ age, height, and body mass are depicted in Table 1. The gymnasts were recruited from a highly reputed training center at Guarulhos, São Paulo. The CRTL group was composed of participants with age-matched to those in the GYN group. The inclusion criteria for the GYN group were being active gymnasts of acrobatic gymnastics currently training for participation in state championships, national league or higher (i.e., pan American and World class), with training routines of at least 15 h/week. The inclusion criteria for CTRL group were no involvement in competitive sports (they typically had their physical activity profile based on physical education classes at school or playing unprofessionally at leisure time). For both groups, exclusion criteria were obesity, musculoskeletal injuries, or vision/neurological disorders that might have affected their ability to maintain balance. At the time of data acquisition, the acrobatic gymnasts were involved in practicing routines 5–6 times per week for 3–5 h (data were acquired 2–3 weeks before the national championship, in which most of them were about to compete). All gymnasts had begun their training experience at least 3 years before the experiments. The experiments were approved by the Human Ethics Committee of the School of Arts, Sciences and Humanities of the University of São Paulo (CAAE69034717.6.0000.5390).

**TABLE 1:** Characteristics (age, height and body mass) of the participants included in the Acrobatic Gymnastics group (GYN, *n* = 14) and age-matched non-gymnasts [controls (CTRL), *n* = 14].

	CTRL	GYN	T_(26)_	P
Age (years)	16.3 ± 3.2 (12–24)	16.1 ± 3.1 (11–23)	0.121	0.905
Body height (cm)	161.4 ± 77.6 (146–174)	156.1 ± 85.6 (147–170)	1.734	0.095
Body mass (kg)	56.7 ± 10.3 (41–74)	53.0 ± 10.3 (30–66)	0.956	0.348

### Electromyography Acquisition

Surface EMG signals were acquired with a DELSYS system, model Trigno-16. EMG signals were captured using surface electrodes positioned (at “preferred” side, corresponding to the support leg in the postural tests, see below) as recommended by SENIAM (Hermens et al., 2000) in *tibialis anterior* (TA), *soleus* (SO), *lateral gastrocnemius* (GL), *medial gastrocnemius* (GM), *vastus lateralis* (VL), and *biceps femoris* (BF) muscles. For the trunk muscles [*spinal erector* (ES) and *rectus abdominis* (RA)], the positioning of the electrodes replicated protocols commonly used in the literature. Thus, RA electrodes were positioned 3 cm laterally to the navel (Horak and Nashner, 1986; Runge et al., 1999; McCook et al., 2009); and in the ES muscle, the electrodes were positioned 3 cm laterally to the spinous process of the third lumbar vertebra (Hodges et al., 2002; McCook et al., 2009). Before electrodes positioning, proper skin preparation was performed, including trichotomy, abrasion and cleaning with alcohol.

### Maximal Isometric Voluntary Contraction Assessments

**FIGURE 1:**
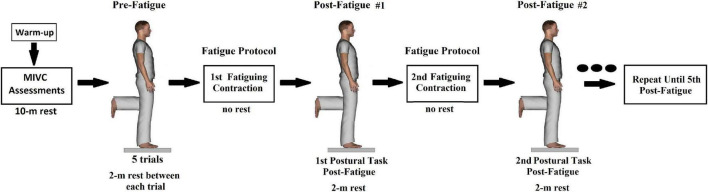
Simplified representation of the experimental protocol.

Figure 1 shows a simplified representation of the experimental procedures. Initially, participants warmed-up on a cycle ergometer for 5 min at low intensity. Then, they performed three maximum isometric voluntary contractions (MIVCs) for each muscle under evaluation, consisting of sustained maximal contractions of approximately 5 s (with verbal encouragement), in which the highest amplitude value of surface EMG (aEMG, see calculation method below) was obtained in a 2-s window. The maximal aEMG observed among the three MIVCs was used to normalize the EMG intensity during the postural tasks (see below). Then, participants had a 10-min rest, seated in a comfortable armchair, before the initial postural assessments.

For MIVC assessments, participants laid (or seated) on a customized stretcher equipped with Velcro tapes and adjustable mechanical levers that prevented undesirable movements of the participants. MIVC of the SO, GL, and GM were obtained with the participants in the prone position, with feet free off the stretcher and ankle at 90°, with the area of the heads of the metatarsal bones in contact with a fixed restraint. Participants were asked to exert as much force as possible to push against the restraint with the foot sole using only leg muscles. A mechanical support was positioned against the participants’ shoulders to prevent them to slip on the stretcher. TA MIVC was obtained with the participants in the supine position, the ankle at 90° and the dorsal part of the foot in contact with a fixed restraint. Participants were asked to pull the restraint with the dorsal part of the foot as hard as possible using only leg muscles. MIVCs of the VL and BF muscles were obtained with the participant seated on the edge of the stretcher with hip, knee, and ankle angles at 90°. A fixed restraint was positioned against the anterior and the posterior aspects of the legs, at ankle level. For the VL, participants were asked to push the anterior restraint forward, with maximum possible force using only thigh muscles, whereas for the BF they were asked to pull the posterior restraint backward. For the RA MIVC, the participants were in the supine position with a fixed restraint positioned over their chest. The participants were asked to exert as much force as possible with only the abdominal muscles in order to try to “lift the restraint.” For the MIVC of the ES, participants were placed in the prone position with a fixed restraint placed over their back (at the level of shoulder blades). Participants were asked to exert the maximum force possible to try to “lift the restraint” using only back muscles. During the MIVCs verbal stimuli were used to motivate participants to exert the maximum possible force.

### Postural Assessments (Pre-fatigue)

Participants were asked to maintain a single-leg stance (unipedal support), with the arms at the side, standing barefoot as quietly as possible over a force plate (OR6-7-1000, AMTI, Watertown, United States). The support leg was the participant’s preferred leg, defined by asking which leg they would use to kick a ball. The knee of the stance leg was in an extended position (but not hyperextended), whereas the hip of the contralateral leg was in a neutral position and the knee was flexed at 90°. Participants looked at a fixed point (i.e., a black circle marked with adhesive tape) at the wall ∼4 m away from them, at eyes level. The position of the subject’s feet on the platform was marked with adhesive tape to ensure that the same position was repeated on each trial. The postural tasks were performed with eyes opened. Each participant performed five trials, each lasting 60 s. A resting period of ∼2 min between trials was allowed (subject sat in a comfortable armchair placed next to the force plate). The single-legged postural task was chosen because it is a posture commonly adopted in the practice of acrobatic gymnastics (present in the punctuation code considered by referees in competitions).

### Fatigue Protocol

Then, plantar flexor muscles of the dominant leg were subjected to muscle fatigue by methodology previously described in the literature (Vuillerme et al., 2001b; Boyas et al., 2013), which consisted of sustained submaximal isometric contractions. The participants were asked to remain in a single-legged posture, “on their toes,” for as long as possible, without performing flexion-extension movements. In other words, the participants had to lift the heel, thereby supporting themselves “on the tip of the foot” with the greatest possible ankle extension by sustaining an isometric plantarflexion force. During the maintenance of this posture, the participants were allowed to lean on a structure positioned in front of them, whenever necessary (i.e., due to any type of imbalance). In addition, an experimenter stayed by the participant’s side during the fatigue protocol, to ensure that no major imbalances occurred. The fatigue condition (i.e., each fatiguing contraction) was considered completed when the participant could no longer maintain this posture “on tiptoe” (i.e., failure, when participants’ heel touched the ground). Verbal stimuli were used to motivate participants to stay until exhaustion. Time to fail was measured [time to failure (in seconds), from the beginning of the fatiguing contraction until failure]. The protocol (fatiguing contraction) was repeated before each trial of single-legged stance performed under the fatigue condition (see below), yielding 5 fatiguing contractions for each participant.

### Postural Assessments (Post-fatigue)

Post-fatigue postural assessments were similar to those described initially (pre-fatigue). However, as a transient effect of fatigue-inducing protocols on postural control has been reported (Paillard, 2012; Kozinc et al., 2021), the fatigue protocol (submaximal contraction until exhaustion, as described above) was repeated before each trial to ensure the participants performed the postural task under the effects of muscle fatigue (Paillard, 2012). For each of the 5 repetitions, the postural task was initiated immediately after the fatiguing contraction was finished, thereby ensuring the presence of the fatigue effect in all post-fatigue trials.

### Data Processing and Analyses

The signals from the EMG and the force plate were acquired by an A/D board (PCI-6015, National Instruments, United States) at 2000 samples/s. Data were analyzed off-line using custom-written programs in Matlab (Math Work Inc., United States).

The EMG signals were pre-amplified and filtered with cutoff frequencies of 20 and 450 Hz. In order to obtain the EMG parameters during the postural tasks, the raw EMG signals were filtered by a 4th-order digital Butterworth bandpass filter (with cutoff frequencies at 20 and 450 Hz). The parameters in the time (EMG amplitude, aEMG) and frequency (median frequency, Fmed) domains were then calculated, with Fmed computed in the frequency spectrum using the Discrete Fourier Transform. For aEMG, the RMS of the EMG signal was computed for the entire period over the postural tasks (60s), for every 250-ms epochs (time window), and averaged among the five trials performed for each condition (i.e., pre and post-fatigue) (Raez et al., 2006; Keenan and Valero-Cuevas, 2008). Then, aEMG values were normalized to the aEMG observed during the MVIC, and presented herein as a percentage of the MVIC, as suggested in the literature (Lehman and McGill, 1999). Muscle activity during MVIC was computed for every 250-ms epochs and averaged. This epoch (250-ms time window) was chosen in order to enhance EMG signal resolution and estimate more accurately aEMG during the postural tasks, which is in line to what has been used in the literature (Raez et al., 2006; Keenan and Valero-Cuevas, 2008). In addition, the same epoch was used in the MVIC to avoid discrepancies in the method of analyzing the data.

The forces and moments measured by the force plate were used to compute the two components of the center of pressure (COP): in the anterior-posterior axis (AP) and the media-lateral axis (ML), indicated as Copa and Comply, respectively. Before analysis, the COP data (acquired at 2 kHz) were low-pass filtered with a digital fourth-order Butterworth filter having an 8 Hz cutoff frequency and the mean was subtracted from each time series. The root-mean-square (RMS) and mean velocity (VM) of the COP data were computed for each axis (i.e., AP and ML). The area of the stabilogram (Area) was estimated from the COP data by fitting an ellipse to the AP x ML COP data that encompasses 95% of the data [using the method proposed by Oliveira et al. (1996)]. The COP velocity was calculated by dividing the total COP displacement (sum of the absolute values of the samples) by the total time interval. All time-domain parameters computed from the COP signals were normalized by participants’ height (Cattagni et al., 2014), and hence are expressed here as a percentage of participants’ height. The power spectral density (PSD) of the COP data (for both AP and ML directions) was estimated in each experimental condition (pre and post-fatigue). The power spectral density was estimated by the Welch periodogram of the detrended data with a resolution of 0.05 Hz. A Hann datawindow was used with subtraction of the best linear regression and a 50% overlap. The area under the PSD was calculated in order to represent the power at two frequency bands: “low frequencies” (LF, 0.05–0.5 Hz) and “high frequencies” (HF, 0.5–2.0 Hz) (Magalhaes and Kohn, 2011). These ranges were chosen as within the adopted low-frequency range (LF), the COP power spectrum approximates that of the center of gravity (Benda et al., 1994), whereas the upper limit of the high-frequency band (2Hz) delimits the frequency range that encompasses 99% of the total power of the COP signal during quiet stance (Mezzarane and Kohn, 2008).

### Statistical Analysis

Participants’ age, height, and body mass were compared between GYN and CTRL groups using *t*-tests for independent samples. COP and EMG parameters (aEMG, Fmed, Area, RMS, VM, HF and LF) were averaged across the 5 repetitions of the postural tasks (for both pre- and post-fatigue assessments). After applying a statistical test to verify the normality of the data (Shapiro-Wilk test), a two-way ANOVA was used to compare the duration of the fatiguing contractions between groups (GYN *vs* CTRL), and among the 5 repetitions (factor “time,” representing the 1^st^ to the 5^th^ fatiguing contraction), with “time” considered as repeated measures. A two-way ANOVA was also used to compare EMG and COP parameters between groups (GYN *vs* CTRL), and between conditions (pre-fatigue *vs* post-fatigue), with this last factor considered as repeated measures. Eventual interactions between factors were obtained by the same test. All the analyses were performed with a significance level set at *p* < 0.05. Effect sizes are reported using partial eta-squared (η^2^_p_) indices (Tabachnick and Fidell, 2007), which indicate the percent of the variance in each of the effects (or interaction) that is accounted for by that effect (or interaction). Effect sizes are interpreted as small (η^2^ = 0.01), medium (η^2^ = 0.06), and large (η^2^ = 0.14) effects.

## Results

### Participants Characteristics

As expected, there were no significant differences between CTRL and GYN for age, as CTRL group were age-matched to GYN. Despite not being matched for body height and body mass, no significant differences between CTRL and GYN were observed for these parameters (see Table 1).

### Time to Failure (Duration of the Fatiguing Contraction)

**FIGURE 2:**
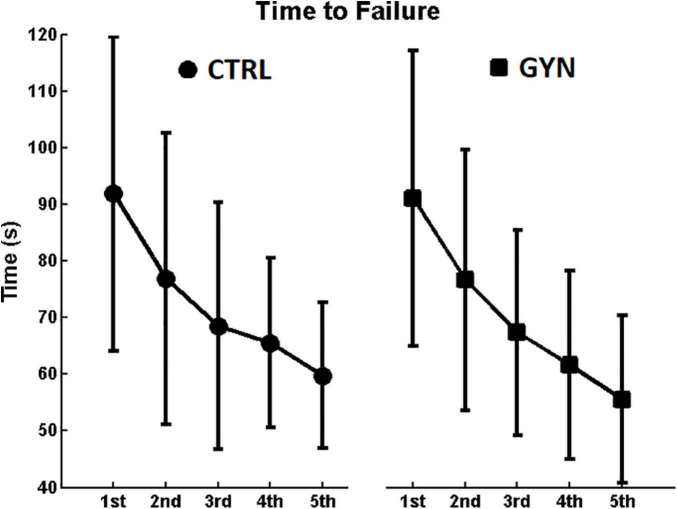
Average duration (time to failure) of each of the 5 fatiguing contraction performed by the participants. Values associated with the controls (CTRL) and gymnasts (GYN) groups are represented by circles (left panel) and squares (right panel), respectively. Note the clear tendency to decrease time to failure (indicating a reduction in endurance performance) along the repetitions, yielding a significant main effect of time (*P* < 0.001). No significant main effect of group was observed neither significant interactions between group and time (*P* > 0.05), indicating that gymnasts showed similar performance during the fatiguing protocol as compared to the controls.

Figure 2 shows the average duration of each of the 5 fatiguing contractions performed by the participants immediately before each of the post-fatigue postural tasks (see methods). A clear tendency to decrease time to failure (indicating a reduction in endurance performance) along the repetitions was observed, as assessed by a significant main effect of time [*F*_(1_,_26)_ = 46.1191; *P* < 0.001; η^2^_p_ = 0.26]. No significant main effect of group was observed [*F*_(1_,_26)_ = 0.0856; *P* = 0.772; η^2^_p_ = 0.0027], neither significant interactions between group and time [*F*_(1_,_26)_ = 0.2284; *P* = 0.922; η^2^_p_ = 0.0017], which indicates that gymnasts showed similar performance during the fatiguing protocol (in terms of duration of the fatiguing contractions) as compared to the controls, along the 5 repetitions of the fatiguing contractions.

### Center of Pressure Analyses (Time and Frequency Domains)

**FIGURE 3:**
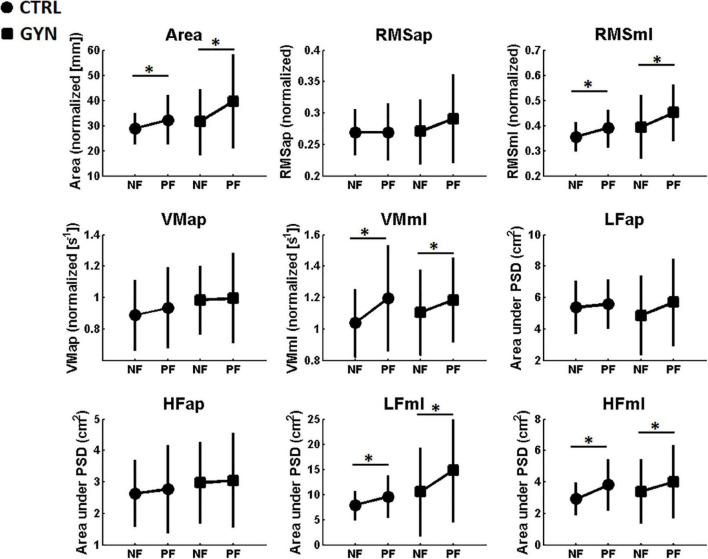
Average center of pressure (COP) measures [associated with the AP (anterior posterior) and ML (medial-lateral) axis] computed before (NF, no fatigue) and after (PF, post-fatigue) the fatiguing protocol. Values associated with the CTRL and GYN groups are represented by circles and squares, respectively. * indicate a statistically significant effect of the fatigue factor, that is, significant differences between NF and PF (*p* < 0.05). Area: area of the area of the stabilogram, RMS: root mean square, VM: COP mean velocity, LF: low frequencies (LF, 0.05–0.5 Hz), HF: high frequencies.

Figure 3 shows the average values of the COP quantifiers (i.e., COP parameters in time and frequency domains) for the CTRL and GYN groups, both before (NF, no fatigue) and after (PF, post fatigue) the fatigue protocol. A significant main effect of fatigue was found for Area, RMSml, VMml, LFml and HFml. These results indicate that the aforementioned parameters were increased after fatigue as compared to pre-fatigue, irrespective of the gymnastic expertise (CTRL *vs* GYN). Therefore, fatigue induced a significant increase in postural oscillations (including velocity and frequency components of the power spectrum), mainly in the ML axis. No significant main effect of fatigue was observed for the parameters that represent the COP exclusively in the AP axis. No significant group effects (CTRL *vs* GYN) were found for COP parameters, neither significant interactions between fatigue and group, which indicates that gymnasts showed similar postural sway as compared to the controls, during both pre and post-fatigue assessments. Table 2 shows the statistical results (F, P, and η^2^) obtained from the two-way ANOVA for the main effects and interactions observed for COP quantifiers.

**TABLE 2:** Statistical results of the two-way analysis of variance (ANOVA) applied to the center of pressure (COP) parameters before and after the fatiguing protocol.

COP	Main effects and interactions								
	Group (CTRL *vs* GYN)			Fatigue (NF *vs* PF)			Fatigue *vs* Group		
	F_(1,26)_	P	η^2^_p_	F_(1,26)_	P	η^2^_p_	F_(1,26)_	P	η^2^_p_
Area	1.3264	0.2599	0.042	10.4663	**0.0033**	0.052	1.6927	0.2047	0.0088
RMSap	0.3883	0.539	0.013	2.114	0.158	0.0099	2.0836	0.161	0.0097
RMSml	2.3033	0.1412	0.069	10.1359	**0.0037**	0.058	0.6227	0.437	0.038
VMap	0.7729	0.3874	0.028	1.9625	0.1731	0.0035	0.8307	0.3704	0.0015
VMml	0.0794	0.7804	0.0029	22.1234	** < 0.001**	0.047	2.4681	0.1283	0.0055
LFap	0.070	0.793	0.0023	2.885	0.101	0.016	1.124	0.298	0.0062
LFml	2.423	0.131	0.079	15.287	** < 0.001**	0.046	2.871	0.102	0.0089
HFap	0.447	0.509	0.015	0.343	0.563	0.0016	0.035	0.851	0.0002
HFml	0.261	0.613	0.0088	11.197	**0.002**	0.047	0.310	0.0014	0.5821

*Significant P values (P < 0.05) are highlighted in bold, indicating significant main effects of fatigue (no fatigue, NF vs post-fatigue, PF). Area: area of the stabilogram, RMS: root mean square, VM: COP mean velocity, LF: low frequencies (LF, 0.05–0.5 Hz), HF: high frequencies.*

### Amplitude of the Electromyography Signal (Electromyography Amplitude)

**FIGURE 4:**
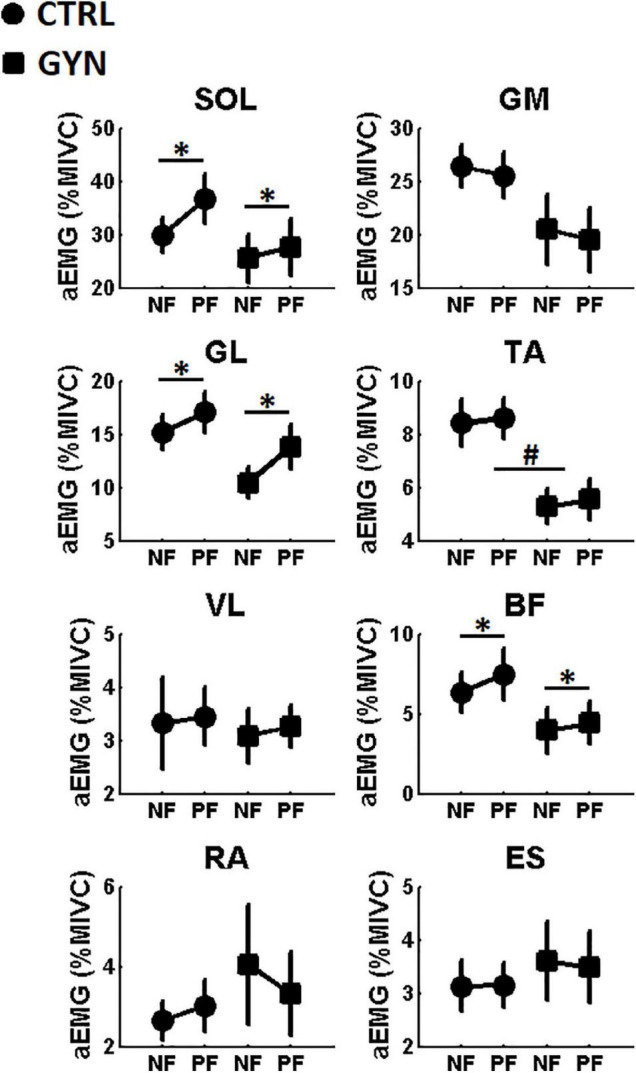
Average amplitude of the EMG signal (aEMG, expressed in% of the Maximal Isometric Voluntary Contraction, MIVC) computed before (NF, no fatigue) and after (PF, post-fatigue) the fatiguing protocol. Values associated with the CTRL and GYN groups are represented by circles and squares, respectively. * indicate a statistically significant effect of the fatigue factor, that is, significant differences between NF and PF (*p* < 0.05). # indicate a statistically significant effect of the group factor, that is, significant differences between acrobatic gymnasts (GYN) and untrained controls (CTRL). TA: *tibialis anterior*, SOL: *soleus*, GL: *lateral gastrocnemius*, GM: *medial gastrocnemius*, VL: *vastus lateralis*, BF: and *biceps femoris*, ES*: spinal erector* and RA: *rectus abdominis*.

Figure 4 shows the average values of aEMG for the CTRL and GYN groups, both before (NF, no fatigue) and after (PF, post fatigue) the fatigue protocol. A significant main effect of fatigue was found for the muscles *soleus* (SO), *lateral gastrocnemius* (GL), and *biceps femoris* (BF). These results indicate that the aEMG recorded from these muscles were significantly increased after fatigue as compared to pre-fatigue, irrespective of the gymnastic expertise (CTRL *vs* GYN). No significant main effect of fatigue was observed for the remaining muscles. A significant group effect (CTRL *vs* GYN) was found for the aEMG of the TA muscle, indicating that the GYN group showed lower TA EMG activation as compared to the CTRL group. No significant main effect of group (CTRL *vs* GYN) was observed for aEMG of the remaining muscles, neither significant interactions between fatigue and group. Table 3 shows the statistical results (F, P and η^2^) obtained from the two-way ANOVA for the main effects and interactions observed for aEMG.

**TABLE 3:** Statistical results of the two-way ANOVAs applied to the measurements of the amplitude of the EMG signal (aEMG) before and after the fatiguing protocol.

aEMG	Main effects and interactions								
	Group (CTRL *vs* GYN)			Fatigue (NF *vs* PF)			Fatigue *vs* Group		
	F_(1,26)_	P	η^2^_p_	F_(1,26)_	P	η^2^_p_	F_(1,26)_	P	η^2^_p_
SOL	1.220	0.279	0.043	9.588	**0.005**	0.019	2.504	0.126	0.005
GM	3.010	0.0946	0.090	0.408	0.528	0.0023	0.001	0.974	<0.0001
GL	3.053	0.0923	0.092	8.265	**0.008**	0.041	0.713	0.405	0.0037
TA	10.055	**0.004**	0.250	0.292	0.593	0.0016	0.013	0.907	<0.0001
VL	0.084	0.774	0.0026	0.164	0.688	0.0012	0.004	0.949	<0.0001
BF	2.003	0.168	0.069	4.365	**0.046**	0.0062	0.852	0.364	0.0012
RA	0.397	0.534	0.014	0.234	0.632	0.0007	2.230	0.147	0.0061
ES	0.260	0.613	0.0094	0.075	0.785	0.0001	0.143	0.707	0.0003

*Significant P values (P < 0.05) are highlighted in bold, indicating significant main effects of fatigue (no fatigue, NF vs post-fatigue, PF) and/or group (control, CTRL vs acrobatic gymnasts, GYN). TA: tibialis anterior, SOL: soleus, GL: lateral gastrocnemius, GM: medial gastrocnemius, VL: vastus lateralis, BF: and biceps femoris, ES: spinal erector and RA: rectus abdominis.*

### Electromyography Median Frequency

**FIGURE 5:**
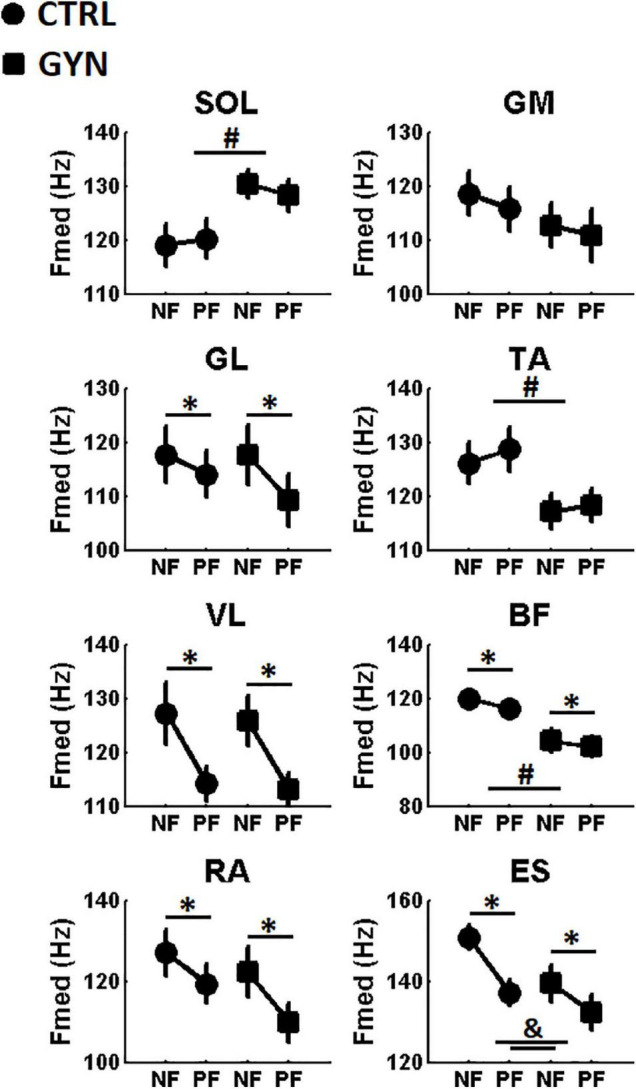
Average Median Frequencies (Fmed, expressed in Hz) computed before (NF, no fatigue) and after (PF, post-fatigue) the fatiguing protocol. Values associated with the CTRL and GYN groups are represented by circles and squares, respectively. * indicate a statistically significant effect of the fatigue factor, that is, significant differences between NF and PF (*p* < 0.05). # indicate a statistically significant effect of the group factor, that is, significant differences between acrobatic gymnasts (GYN) and untrained controls (CTRL). & indicate significant interaction between “group” and “fatigue period” factor. & indicates a statically significant effect for “group” factor (*p* < 0.05). TA: *tibialis anterior*, SOL: *soleus*, GL: *lateral gastrocnemius*, GM: *medial gastrocnemius*, VL: *vastus lateralis*, BF: and *biceps femoris*, ES*: spinal erector* and RA: *rectus abdominis*.

Figure 5 shows the average values of Fmed for the CTRL and GYN groups, both before (NF, no fatigue) and after (PF, post fatigue) the fatigue protocol. A significant main effect of fatigue was found for the muscles *lateral gastrocnemius* (GL), *vastus lateralis* (VL), *biceps femoris* (BF), *rectus abdominis* (RA) and *spinal erector* (ES). These results indicate that the Fmed of the EMG recorded from these muscles were decreased after fatigue as compared to pre-fatigue, irrespective of the gymnastic expertise (CTRL *vs* GYN). No significant main effect of fatigue was observed for the remaining muscles. A significant group effect (CTRL *vs* GYN) was found for the Fmed of the SOL, TA and BF muscles, indicating that the GYN group showed higher Fmed in SOL EMG signal, but lower Fmed in TA and BF EMG signals as compared to the CTRL group. No significant main effect of group (CTRL vs GYN) was observed for the remaining muscles. Interestingly, a significant interaction between fatigue and group was observed for Fmed of the ES muscle, indicating a less pronounced effect of fatigue (i.e., decreased Fmed) for the GYN group as compared to the CTRL group. No significant interactions between fatigue and group were found for Fmed of the remaining muscles. Table 4 shows the statistical results (F, P, and η^2^) obtained from the two-way ANOVA for the main effects and interactions observed for Fmed.

**TABLE 4:** Statistical results of the two-way ANOVAs applied to the measurements of the median frequency of the EMG signal (Fmed) before and after the fatiguing protocol.

Fmed >	Main effects and interactions								
	Group (CTRL *vs* GYN)			Fatigue (NF *vs* PF)			Fatigue *vs* Group		
	F_(1,26)_	P	η^2^_p_	F_(1,26)_	P	η^2^_p_	F_(1,26)_	P	η^2^_p_
SOL	4.681	**0.039**	0.150	0.195	0.661	0.0003	2.641	0.116	0.0051
GM	0.901	0.351	0.032	3.242	0.083	0.0064	0.127	0.724	0.0003
GL	0.132	0.718	0.005	12.869	**0.001**	0.0280	1.985	0.170	0.0044
TA	4.235	**0.049**	0.130	4.096	0.060	0.0053	0.643	0.429	0.0007
VL	0.0535	0.818	0.002	24.675	** < 0.001**	0.150	0.0041	0.948	<0.0001
BF	11.103	**0.002**	0.290	8.983	**0.006**	0.015	0.584	0.451	0.001
RA	1.028	0.320	0.034	17.225	** < 0.001**	0.066	1.010	0.324	0.0042
ES	2.573	0.120	0.083	46.855	** < 0.001**	0.130	4.753	**0.038**	0.015

*Significant P values (P < 0.05) are highlighted in bold, indicating significant main effects of fatigue (no fatigue, NF vs post-fatigue, PF) and/or group (control, CTRL vs acrobatic gymnasts, GYN) and/or significant interactions between fatigue and group. TA: tibialis anterior, SOL: soleus, GL: lateral gastrocnemius, GM: medial gastrocnemius, VL: vastus lateralis, BF: and biceps femoris, ES: spinal erector and RA: rectus abdominis.*

## Discussion

The present study compared the performance of single-legged stance and the associated modulation of time and frequency-domain EMG parameters between trained gymnasts and healthy untrained controls before and after a protocol designed to induce muscle fatigue in the ankle plantarflexor muscles. As hypothesized, fatigue-induced increased postural sway, while decreasing Fmed and increasing aEMG, irrespective of the gymnastics expertise. We had also hypothesized that fatigue-induced effects on COP and EMG parameters would be more pronounced in the non-trained group as compared to GYN, as the trained gymnasts would have counteracted (at least partially) the deleterious effects of fatigue by using neuromuscular adaptations developed as a result of their intense and regular practice. This last hypothesis was only partially confirmed (i.e., only confirmed in terms of EMG activation), as no superior stability was found for the GYN group as compared to CTRL in terms of postural sway parameters (i.e., COP quantifiers), irrespective of the fatigue condition (see Figure 3). On the other hand, expert acrobatic gymnastics used different neuromuscular control strategies to keep their postures on single-legged quiet standing [i.e., with lower aEMG of the TA muscle (see Figure 4), higher Fmed of the SO muscle, and lower Fmed of TA and BF muscles (see Figure 5)]. Notable, a more pronounced effect of fatigue on the ES muscles (i.e., decreased Fmed) was observed for the CTRL group as compared to the GYN group.

The fatigue protocol (submaximal contractions until exhaustion) was repeated before each trial associated with the post-fatigue condition to warrant that participants performed the postural assessments still under the effects of muscle fatigue (Paillard, 2012). The duration of the fatiguing contractions significantly decreased along with the repetitions (see Figure 2), irrespective of gymnastics expertise, indicating that the endurance performance of the ankle plantarflexor muscles tended to decrease with repetition, thereby suggesting the fatigue protocol was effective. Additionally, significant increases in aEMG values and significant decreases in Fmed values were observed after the fatigue protocol (see Figures 4, 5) as compared to baseline assessments, which are in accordance with the most pertinent literature (Merletti et al., 1991; Lowery et al., 2002; Mathur et al., 2005), as mentioned in the Introduction section. Altogether, these results indicate that the fatigue protocol used in the present study proved to be effective, representing an appropriate methodology for the comparisons between experimental conditions. Further analyses regarding time and frequency-domain EMG parameters recorded during the fatiguing protocol were previously published as a conference paper (Silva et al., 2021).

The duration of the fatiguing contractions was not significantly different between groups, and no significant interactions between group and time were observed, which indicate that gymnasts showed similar performance during the fatiguing protocol (in terms of duration of the fatiguing contractions) as compared to CTRL, along with the 5 repetitions of the fatiguing contractions. Therefore, all putative mechanisms associated with the differential effects of fatigue on COP and EMG measurements (discussed below) between GYN and CTRL are very probably associated with the gymnastics expertise itself, rather than a mere consequence of a different fatigue protocol.

Center of pressure (COP) analyses showed that fatigue induced a significant increase in postural oscillations in the ML axis, with no significant effects in the AP axis, whereas no superior stability was found for the GYN group as compared to CTRL in terms of postural sway parameters (i.e., COP quantifiers), irrespective of the fatigue condition (see Figure 3). These results are in line with previous studies that reported no significant differences in some postural sway parameters between gymnasts and non-gymnasts (Vuillerme et al., 2001a; Asseman et al., 2004, 2008; Vuillerme and Nougier, 2004; Gautier et al., 2008; Opala-Berdzik et al., 2021). As these studies used relatively non-challenging postural tasks (bipedal eyes-open posture), it has been suggested that the effect of gymnastics expertise on balance ability might be associated with specific postures for which the practice is specifically related (Asseman et al., 2008), as gymnasts’ training routines (which involve difficult and specific postures) might have a slight or no effect on the performance of unspecific postures. Thus, in the present study, we chose the unipedal stance (performed with eyes open) as the postural task because it represents a more challenging and specific posture associated with acrobatic gymnastics training. However, unlike previous studies that reported significant differences between gymnasts and controls on some postural sway parameters for this condition (Asseman et al., 2008), our study did not find superior balance stability for the GYN. These different results might be associated with the different samples used in the studies, as Asseman et al. (2008) investigated male artistic gymnasts (and compared them with other sportsmen), whereas the present study investigated younger female expert acrobatic gymnastics as compared to non-trained counterparts. Indeed, expertise (Opala-Berdzik et al., 2021), sex (Milosis and Siatras, 2012) and training specificity (Vuillerme et al., 2001a; Asseman et al., 2004, 2008; Isableu et al., 2017) have been shown to significantly influence the development of postural control abilities.

As expected, fatigue induced a significant increase in postural oscillations, irrespective of the gymnastics expertise. Interestingly, such an effect was only observed for the postural parameters computed for the ML axis (as no significant main effect of fatigue was observed for the parameters that represent the COP exclusively in the AP axis). This might be related to the nature of the unipodal balance task used in the present study, in which the body oscillations occur mainly along the ML axis. Calavalle et al. (2008) showed superior balance stability during quiet bipedal posture in rhythmic gymnasts as compared to controls trained in other sports, which only occurred in the ML direction. However, these results cannot be directly compared as they relied in different samples and postures, and the effect of fatigue was not explored in the study of Calavalle et al. (2008).

A previous study (van Dieen et al., 2012) showed that fatigue (i.e., induced by exercising trunk muscles) negatively affected trunk stability in elite gymnasts (for both unperturbed balancing and recovery after balance perturbation), but no comparisons were made with other groups so that we cannot speculate whether gymnasts expertise might be associate with a differential ability in dealing with the detrimental effects of fatigue on trunk stability. To the best of our knowledge, the present study is the first to investigate the fatigue-related effects on unipedal postural control of expert acrobatic gymnasts as compared to untrained matched controls. Contrary to our initial hypothesis, the effects of fatigue in reducing balance steadiness during the single-legged stance task were comparable between GYN and CTRL (i.e., neuromuscular adaptations due to acrobatic gymnastics training was not able to significantly counteract for the detrimental effects of fatigue), at least regarding the COP-related postural sway measurements explored in the present study. Isableu et al. (2017) showed that non-linear dynamical analysis of COP trajectories might be useful to provide a deeper understanding of the mechanism behind sports-related adaptations on postural control mechanism, as the analysis of the COP regularity (i.e., sample entropy measurements) could distinguish between the bipedal quiet stance performance of gymnasts and non-gymnasts, which could not be evidenced by the traditional sway parameters. Therefore, future studies should focus on deeper analyses of postural sway parameters to explore whether gymnastics expertise might be associated with the differential ability to deal with muscle fatigue.

The comparisons between the EMG parameters recorded before and after the fatigue protocol indicate that the effect of fatigue was not restricted to the target muscles (plantar flexors), considering that, except for SOL, GM, and TA, all Fmed values decreased significantly after the fatigue protocol (which includes tight and trunk muscles) and aEMG of the BF muscle significantly increased after the fatigue protocol (besides SOL and GM). These results reinforce the notion that the fatigue effect associated with a specific contraction might be extended beyond the target musculature (McDonald et al., 2019; Goethel et al., 2020). This has important implications from a practical standpoint as it suggests that the performance of different gestures might be impaired after the execution of a task with little or no similarity to the one that induced fatigue. For instance, coaches, athletes, and referees must be aware that there might be a reduction in performance (and probably an increased injury risk) induced by a fatigue process generated by a previous exercise/routine, even when the task performed might seem fairly unrelated to the previous one.

Acrobatic gymnastics showed lower TA aEMG as well as lower TA and BF (but higher SOL) Fmed as compared to the CTRL group. Finally, it is worth noting that the Fmed of the ES muscle decreased more sharply after the fatigue protocol for the CTRL group (evidenced by the significant interaction between the factors “fatigue” and “group,” see Figure 5 and Table 4), indicating a greater effect of fatigue of the ES for the CTRL group as compared to the GYN group. These results suggest that trained acrobatic gymnasts develop specific neuromuscular strategies to deal with balance maintenance on single-legged stance (albeit these differential strategies seem to not influence their postural oscillations, at least in terms of the COP parameters analyzed herein). Higher levels of TAEMG activity during postural tasks have been traditionally associated with less stable situations, such as during the control of quiet bipedal stance performed by older adults (as compared to younger ones) (Kouzaki and Shinohara, 2010), which probably reflects more frequent corrections on sway trajectory to keep the body still. Therefore, the lower EMG activation (aEMG) of the TA muscle shown by the gymnasts as compared to CTRL suggests a training-induced adaptation that led the athletes to a reduced need to activate the anterior leg muscles during the control of single-legged stance (which also seems to be reflected by the lower TA Fmed). From a practical standpoint, the changes in the patterns of EMG activation shown by the acrobatic gymnasts as compared to their counterparts suggest that the design of training strategies might focus on increasing the strength, endurance, and control capacities of specific muscle groups that include the *tibialis anterior*, *soleus*, *biceps femoris*, and *spinal erector* muscles. The rationale relies on the preparation of specific muscle groups differentially used by the gymnasts to keep their postures on single-legged quiet standing before and after the fatiguing protocol. However, the potential benefit of such strategies in improving the athletes’ performance and/or reducing injury risk is made here in a speculative way, so that appropriated controlled trials might be undertaken to test this hypothesis.

The present study included participants aged between 11 and 24 years. It is well known that the performance of the postural control system changes across the lifespan (Barela et al., 2003; Peterson et al., 2006; Kurz et al., 2018; Schedler et al., 2019), so that balance adaptability might be differentially developed in children, adolescents, and adults. Despite the majority of our sample comprised adolescents between 14 and 19 years old (only 1 participant in each group was 11–12 years old and other 23–24 years old), we cannot rule out the possibility that the wide range of participants’ age has influenced the results somehow, as some of them might have had their postural performance associated with specific developmental changes in the postural control system.

Altogether, these results indicate that acrobatic gymnastics athletes used different neuromuscular control strategies (as evidenced by the EMG parameters in time and frequency domains), to keep a balance steadiness during the single-legged stance performed before and after the fatigue protocol. Further studies are warranted to investigate whether these mechanisms are accompanied by differences in the angular variation of specific joints (e.g., ankle, knee, hip) that may not have been reflected on the EMG parameters, as well as any differences in the coordination patterns of the associated joint and segments with respect to each other.

## Conclusion

This study investigated whether expert acrobatic gymnasts respond differentially than their non-trained counterparts in controlling single-legged stance task performed before and after a protocol designed to induce fatigue in the ankle plantarflexors muscles (i.e., fatigue achieved by maintaining the posture “on the tip of the toes”). Fatigue induced a significant increase in postural oscillations in the ML axis, with no significant effects in the AP axis. In terms of postural sway parameters (i.e., COP quantifiers), no superior stability was found for the GYN group as compared to CTRL, irrespective of the fatigue condition. On the other hand, the modulation of EMG parameters (in both time and frequency domains) indicated that expert acrobatic gymnastics athletes (as compared to healthy untrained matched controls) used different neuromuscular control strategies to keep their postures on single-legged quiet standing before and after the fatiguing protocol. Future studies are needed to investigate whether such mechanisms are associated with different movement patterns or, alternatively, are manifested only in the electrical patterns of muscle activation. The efficacy of strategies aimed at improving the performance of specific muscles (i.e., *tibialis anterior*, *soleus*, *biceps femoris* and *spinal erector*, for which particular activation patterns were used by the acrobatic gymnastics to control single-legged quiet standing) on gymnasts’ performance must be investigated by appropriate controlled trials.

## Data Availability Statement

The raw data supporting the conclusions of this article will be made available by the authors, without undue reservation.

## Ethics Statement

The studies involving human participants were reviewed and approved by Human Ethics Committee of the School of Arts, Sciences and Humanities of the University of São Paulo (CAAE69034717.6.0000.5390). Written informed consent to participate in this study was provided by the participants’ legal guardian/next of kin.

## Author Contributions

FM and CS were equally involved in the conceptualization and design of the study. MS recruited participants. MS, FL, JG, and JL managed data collection. FM, FL, and CS completed data processing and analysis. FM drafted the first version of the manuscript and supervised data collection. All authors assisted with drafting, provided critical revision of the manuscript, and read and approved the final version of the manuscript.

## Conflict of Interest

The authors declare that the research was conducted in the absence of any commercial or financial relationships that could be construed as a potential conflict of interest.

## Publisher’s Note

All claims expressed in this article are solely those of the authors and do not necessarily represent those of their affiliated organizations, or those of the publisher, the editors and the reviewers. Any product that may be evaluated in this article, or claim that may be made by its manufacturer, is not guaranteed or endorsed by the publisher.
